# New insights on mucormycosis and its association with the COVID-19 pandemic

**DOI:** 10.2144/fsoa-2021-0122

**Published:** 2021-12-16

**Authors:** Mona G Alshahawey, Ghadir S El-Housseiny, Noha S Elsayed, Mohammad Y Alshahrani, Lamia M EL Wakeel, Khaled M Aboshanab

**Affiliations:** 1 Department of Clinical Pharmacy, Faculty of Pharmacy, Ain Shams University, Cairo, 11566, Egypt; 2 Department of Microbiology & Immunology, Faculty of Pharmacy, Ain Shams University, Cairo, 11566, Egypt; 3 Department of Clinical Laboratory Sciences, College of Applied Medical Sciences, King Khalid University, Abha, 9088, Saudi Arabia

**Keywords:** COVID-19, epidemiology, fungal infection, host immune response, mucormycosis

## Abstract

COVID-19 continues to cause significant fatality worldwide. Glucocorticoids prove to play essential roles in COVID-19 management; however, the extensive use of steroids together with the virus immune dysregulation may increase the danger of secondary infections with mucormycosis, an angioinvasive fungal infection. Unfortunately, a definite correlation between COVID-19 and elevated mucormycosis infection cases is now clear worldwide. In this review, we discuss the historical record and epidemiology of mucormycosis as well as pathogenesis and associated host immune response, risk factors, clinical presentation, diagnosis and treatment. Special emphasis is given to its association with the current COVID-19 pandemic, including latest updates on COVID-19-associated mucormycosis cases globally, with recommendations for efficacious management.

## Lay abstract

A definite correlation between COVID-19 and elevated mucormycosis infection cases is now clear worldwide. This article can be used as comprehensive tool to help clinicians and the healthcare team in getting a clearer look on the historical record and epidemiology of mucormycosis as well as pathogenesis and associated host immune response. Risk factors, clinical presentation, diagnosis, treatment options and also the latest updates on COVID-19-associated mucormycosis are also discussed, which will help in the understanding of the fatal mucormycosis infection.

The whole world has experienced a pandemic due to COVID-19 caused by SARS-CoV-2. Since day 1 of its detection in December 2019 in Wuhan, China, and up to the present, various twists and turns in terms of disease pathophysiology, diagnosis, treatment and, most important, its sequelae and complications are being revealed. The COVID-19 symptom spectrum is continuously expanding ranging from initial dry cough and high-grade fever, to multisystem dysfunction that may eventually end with death [1].

Otorhinolaryngology has been intensively involved in COVID-19 diagnosis and management, starting from diagnosis by nasopharyngeal swab sampling or anosmia as a typical symptom marker, to the detection of virus isolates from the middle ear [2]. A recent, more dangerous association between ear, nose and throat pathology and COVID-19 was noticed by the detection of cases with mucormycosis-associated fungal sinusitis. The cases have been reported during the disease course or as a disease sequela. Generally, invasive fungal infections are believed to be rare. In a case–control study from 2006 to 2019, Larcher *et al.* reported 6000 critically ill patients admitted to the intensive care unit, with only 26 patients having invasive fungal infections. Of these 26 patients, half were infected with mucormycosis [3]. Although mucormycosis infections are rare, they are associated with high mortality rates, ranging from 40 to 80% [4,5]. As we are experiencing a resurgence of mucormycosis infection in the era of COVID-19, there is an urgent need to develop new strategies for better treatment and prevention. COVID-19-associated mucormycosis in now creating an epidemic in a global pandemic, particularly in the low- to middle-income countries [6], where poor management, late diagnosis and misleading treatment plans continue to occur. This article maybe used as comprehensive tool to help clinicians and healthcare team getting clearer look on the risk factors, clinical presentation, diagnosis, treatment options and the latest updates on COVID-19 associated mucormycosis, helping them reduce the burden of the fatal mucormycosis infection.

## Mucormycosis

Mucormycosis is caused by Mucorales order, which is widely distributed in the environment, found in, for example, decaying food and air-conditioning filters [7]. However, soil is the main habitat for this fungus. Moreover, Mucorales species are diverse across different countries. For example, *Lichtheimia* species are widespread in Europe and completely absent from the Americas [8]. Mucormycosis is also called zygomycosis or phycomycosis, and it is known misleadingly as ‘black fungus’, perhaps because of it may induce tissue necrosis, which eventually tends to be black in color [9]. However, Mucorales actually lack melanin in their cell wall, and ‘black fungi’ are related to different category [10]. Zygomycosis was initially described in Germany back in 1876 when Fürbinger reported the death of a cancer patient whose right lung suffered a hemorrhagic infarct with fungal hyphae and sporangia [11].

The first reported case of disseminated mucormycosis, termed ‘mycosis mucorina,’ was published in 1885 by Arnold Paltauf [12]. Later, in 1957, it was called as ‘mucormycosis’ by an American pathologist, Baker [13], and described as an invasive fungal infection caused by *Rhizopus*. Additional cases then appeared, and the disease incidence gradually increased [14]. Mucorales fungi currently rank as the second most widespread mold pathogens after *Aspergillus*, causing invasive fungal disease in malignancy, after transplantation or in diabetes [15,16], the latter being the most common predisposing factor universally.

## Taxonomy

Taxonomically, Mucorales classification has not been consistent over the years. In the past, the phylum Zygomycota comprised the Mucorales, Entomophtorales and others nonhuman pathogens [17]. Zygomycota fungi reproduce sexually and produce zygospores, which are formed after fusion of hyphal ends and result in a thick-walled and pigmented zygote [18]. With the advancement in molecular methods of detection, a new classification was used, and Zygomycota was abandoned because it consists of different taxa and groups satisfying its definition [19]. In 2012, the term ‘zygomycosis’ was substituted by either ‘mucormycosis’ or ‘entomophthoromycosis’ [20]. Later in 2016, Spatafora *et al.* carried out a phylogenetic analysis and concluded that Zygomycetes include two phyla; Mucoromycota and Zoopagomycota. The Mucoromycota included Glomeromycotina, Mucorales, and Mucoromycotina [21].

The genera of the Mucorales consist of 261 species, only 38 of which cause human infections [18]. The most important clinical species of these genera are listed in Table 1. The most common type is reported to be *Rhizopus arrhizus* [22].

**Table 1.** Most important clinical species of Mucorales.

Genus	Species	Ref.
*Lichtheimia*	*L. corymbifera* *L. ramosa* *L. ornata*	[23]
*Mucor*	*M. circinelloides*	[24]
*Rhizomucor*	*R. pusillus* *R. miehei*	[25]
*Rhizopus*	*R. arrhizus*, *R. microsporus* *R. homothallicus* *R. schipperae*	[18,26]
*Cunninghamella*	*C. bertholletiae* *C. blakesleeana* *C. echinulata* *C. elegans*	[18,27]
*Saksenaea*	*Saksenaea vasiformis*	[9]

## Epidemiology

Mucormycosis epidemiology develops with the appearance of new strategies in immunotherapy for cancer and autoimmune diseases, together with novel diagnostic methods that aid in the identification of a formerly uncommon species. For example, *Saksenaea erythrospora* is a recently described species causing mucormycosis; PCR amplification and internal transcribed spacer sequencing are the gold standards for its identification [28]. Case reports and case series are the source of most of the information forming the epidemiology of mucormycosis. In 2005, Roden *et al.* published the first extensive analysis [29], which included 929 cases reported in the period 1940–2003. The review, although informative, included entomophthoramycosis cases. Jeong *et al.* later published a review using the PRISMA guidelines and including mucormycosis cases solely amounting to 851 cases reported in the period 2000–2017 [30]. Case series are limited by being obtained on a national level [31] or only in patients with certain diseases [32]. Another valuable source are registries, such as that constructed by the Working Group on Zygomycosis of the European Confederation of Medical Mycology (ECMM) and the International Society of Human and Animal Mycology (ISHAM) in 2004 (www.zygomyco.net), which published 230 case reports from Europe in 2011 [33].

The incidence of mucormycosis is rising internationally. A study in the USA documented an increase in the mucormycosis incidence in hematological malignancy patients from 0.006 cases/100 autopsies in 1989–1993 to 0.018 cases in 2004–2008 [34].

Prevalence has also been rising in Europe. A center in Switzerland reported an increased prevalence from 0.57 cases/100,000 admissions/year before 2003 to 6.3 cases/100,000 admissions/year after 2003, as a result of excessive voriconazole and caspofungin use [35]. In a population-based study in France, the prevalence rose from 0.7 cases/million in 1997 to 1.2/million in 2006 [31]. In Belgium, a rise from 0.019 cases/10,000 patient-days in 2000 to 0.148 cases/10,000 patient-days in 2009 was reported [36].

In Asia, a comparable increase in prevalence was stated in several studies. A retrospective study from Iran including 208 mucormycosis cases from 2008 to 2014 demonstrated a substantial rise from 9.7% in 2008 to 23.7% in 2014 [37,38]. A national survey on medical autopsies conducted in Japan reported an increase in Mucormycosis from 0.01% cases in 1969 to 0.16% cases in 1989 [39]. India reported an increase in mucormycosis cases from 24.7 cases per year (1990–2007) to 89 cases per year (2013–2015) at a single tertiary-care hospital [40]. The escalation of mucormycosis in India was also demonstrated by Chakrabarti *et al.* who issued 3 successive studies from the same center. Prevalence rose from 12.9 cases/year during 1990–1999 [41] to 35.6 cases/year during 2000–2004 [16] and then to 50 cases/year during 2006–2007 [42].

Scarce data is known about mucormycosis prevalence in the Arabian countries [43]. An annual rate of 0.2 cases/100,000 individuals has been reported in Iraq, Jordan and Algeria, and 1.2 cases/100,000 individuals were reported in Qatar [44,45]. Eighteen patients were recognized in another retrospective study carried out at a tertiary care center in the Kingdom of Saudi Arabia from January 2013 to December 2019 [46]. In Lebanon, incidence has significantly risen from 0.47 cases/10,000 admissions in 2008 to 1.18 cases/10,000 admissions in 2017 [47]. From Egypt, Zaki *et al.* reported 10 cases of mucormycosis, all detected at the Ain Shams University Specialized Hospital in Cairo, Egypt, during 2010 [48]. Another retrospective study was carried out at the Children’s Cancer Hospital, Cairo, Egypt, during 2007–2017 and recorded 3.2 cases/1000 pediatric cancer patient admission; 90% of the cases had hematological malignancies [49].

Recently, an article was published including analyzed cases of mucormycosis across the Middle East and North Africa region. Cases of proven or probable invasive mucormycosis were recognized from the FungiScope database and the medical literature. A total of 310 cases of mucormycosis were reported mostly from Iran (n = 74), Israel (n = 63) and Tunisia (n = 49). Others were reported from Lebanon (n = 28), Saudi Arabia (n = 28), Egypt (n = 20), Iraq (n = 11) and Qatar (n = 10), and other countries reported fewer than 10 cases. Reported cases rose from 23 before 1990 to 127 in the 2010s [50]. Lack of data in our region makes it difficult to compare the mucormycosis epidemiology to global studies. However, a few countries, such as Lebanon, are currently partaking in international registries on mucormycosis overseen by the ECMM, which is valuable in comparing epidemiological and clinical variations among different regions [43].

In a comparison between the economies of the countries affected by mucormycosis, it was found, surprisingly, that high-income countries have an higher odds ratio for mortality than low-income countries. Looking into this observation revealed that the rare nature of mucormycosis in developed countries has led to fewer physicians being trained to manage this disease differently from other, similar diseases. Moreover, physicians from low-income countries may be more likely to report patients who survive than deceased cases [51].

Difficulty in sample collection from deep tissues and low sensitivity of diagnostic tests leads to many cases remaining undiagnosed; thus, the actual prevalence of mucormycosis may be greater than that reported. According to the Leading International Fungal Education portal, which estimates the load of serious fungal infections worldwide, the yearly prevalence of mucormycosis may be ~10,000 cases worldwide, excluding India. The estimate rises to 910,000 cases globally if India is included in the data [52,53]. The predicted incidences per million inhabitants on diverse continents were as follows: Europe (from 0.2 cases in Denmark to 95 cases in Portugal), USA (3.0 cases), Canada (1.2 cases) and Australia (0.6 cases) [54]. A computational-based method approximated the prevalence at 140 cases/million populations in India [53]. This reveals that the estimated prevalence of mucormycosis in India is ~70 times higher than the rest of the world. All the preceding reports emphasize that mucormycosis is an evolving disease [55].

## Pathogenesis & host immune response

Generally, the Mucorales can enter the host either through inhalation, percutaneous inoculation or ingestion [9]. Mucorales are saprophytic fungi with a ubiquitous distribution; they can be found in soil, air and food [56]. Surprisingly, they can be present in the nasal mucosa of healthy individuals as a commensal [57]. However, when patients become immunosuppressed [58], the fungus may germinate and migrate through the paranasal sinuses, spread intracranially and reach the nearby structures, such as orbitals. Moreover, the damage caused to the endothelial cells allows the fungal angioinvasion and incident vessel thrombosis with subsequent tissue necrosis [59].

Mucorales must scavenge ample iron from hosts to grow and evade the host’s phagocytic defense mechanisms [60]. They gain access to vasculature via attachment to extracellular matrix proteins and adherence to endothelial cells, so that they can disseminate and spread [61,62]. Both observational and experimental evidence have pointed unequivocally to phagocytes as the primary defense for the host [60]. That's why neutropenic patients and those with defective phagocytes are believed to be most susceptible to develop severe invasive mucormycosis [63,64]. Mucormycosis tends to affect immunocompromised individuals [60] and those with profound neutropenia [4].

The body faces any infection through the cooperative interplay of the innate and adaptive immune system. The innate immune system is the first nonspecific host response against pathogens. It is composed mainly of physical barriers (skin, alveoli or gut) and immune effector cells (macrophages, neutrophils, NK cells and dendritic cells). The adaptive immune system is the second line of defense against pathogens and is composed of T and B cells. The severity of mucormycosis is mainly due to the failure of the immune system to fight those fungi [65].

**Figure 1.**
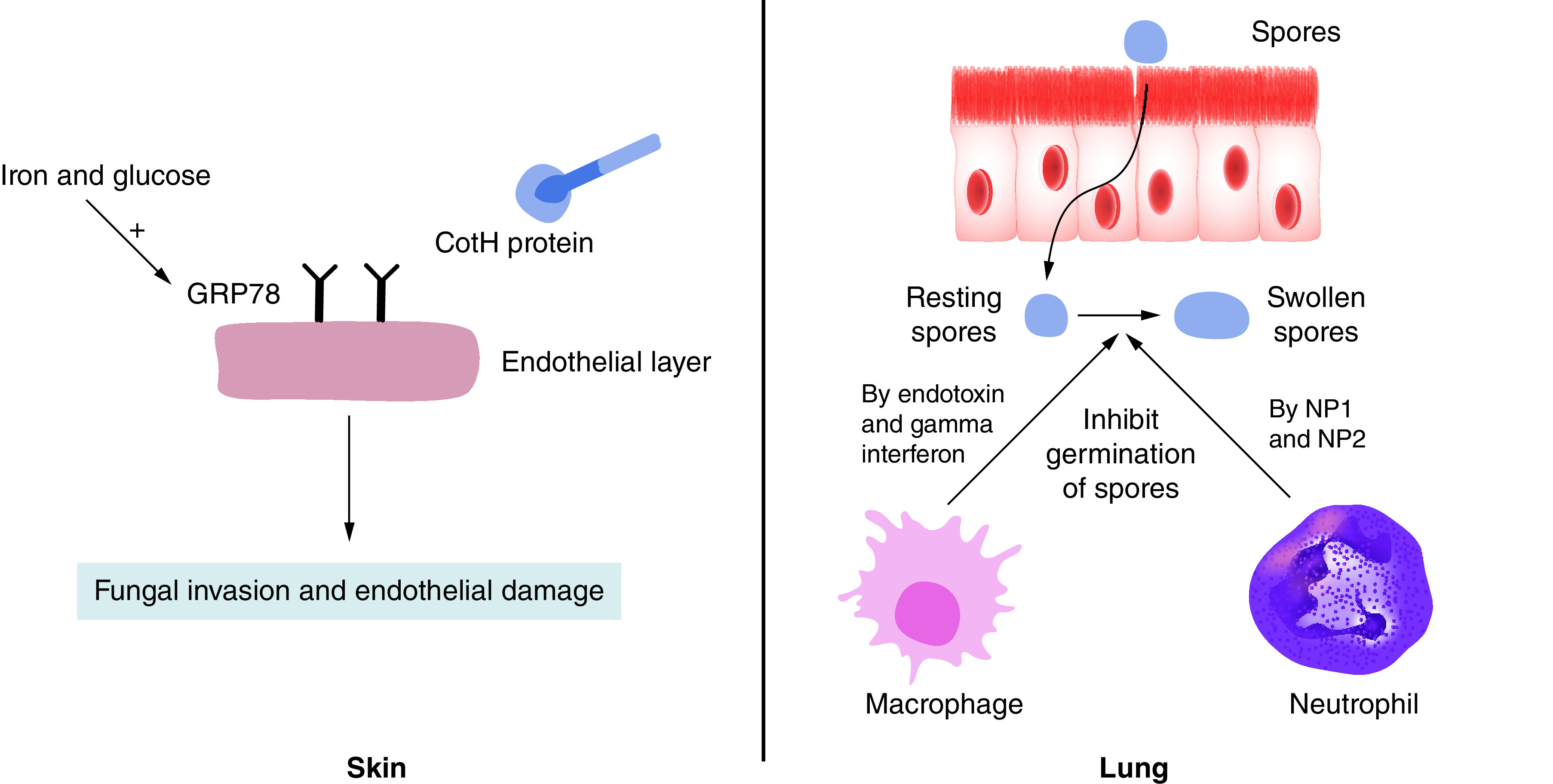
Mechanism of entry of Mucorales through the skin and body immune response against respiratory tract entry.

Initially the Mucorales cross the skin through the wounds and gut through ingestion, thus they successfully evade the physical barriers of the body [66,67]. The most susceptible patients for invasive mucormycosis mostly have epithelial damage especially in the basement membrane. This damage being often caused by diabetes or chemotherapy, exposes the extracellular protein matrix where the fungal spores adhere to its components (lamillin and collagen IV) [68]. The binding fungal ligand is the spore coating (CotH) protein family which is found only in Mucorales. It binds to the host receptor glucose regulator protein 78 (GRP78) and induces the endothelial cell-mediated fungal endocytosis (Figure 1).

Glucose and iron increase the expression of GRP78 [69] in diabetic ketoacidosis (DKA) patients which amplifies their susceptibility to mucormycosis [70]. Iron levels are high in DKA patients because of the hyperglycemia-induced glycosylation of iron binding proteins such as ferritin, which in turn decrease their affinity to bind iron [68]. On the other hand, surprisingly, the administration of deferoxamine, an iron chelator, for hemodialysis or thalassemic patients to decrease the iron load was reported to decrease the survival of infected guinea pigs [71]. It was found that either *Rhizopus* allows the entry of the deferoxamine iron complex via siderophore shuttle mechanism [72] or *Rhizopus* uptakes the iron from the deferoxamine iron complex through an energy-dependent process using reductase enzyme to convert ferric to soluble ferrous [68]. In a nutshell, the elevated levels of glucose and iron in DKA and deferoxamine-treated patients induce the GRP78 expression and binding to CotH proteins, leading to fungal invasion and the successive endothelial injury.

Another entry for Mucorales is the respiratory tract, where the alveolar macrophages are the first line of defense (Figure 1). Macrophages’ role in the innate immune system in general is to recognize infection early and present its antigens to the adaptive immune system. The macrophages respond to fungal infection by inhibiting the germination of spores or conidia. Waldrof *et al.* reported the effect of macrophages in healthy mice infected with *Rhizopus*, the absence of spore or conidia germination in histology samples of lung tissue. However, they observed that the spores remain viable for 10 days in the lung. Thus, they concluded that bronchoalveolar macrophages inhibit the germination of *Rhizopus* spores but were unable to kill them [73]. Jornes *et al.* revealed the mechanism of inhibition of spore germination by macrophages through production of endotoxin and gamma interferon [74]. In case of *Lichtheimia corymbifera*, the macrophages’ role was totally unexpected and surprising. Through fluorescent microscopy, it was found that the phagocytosis ratio was higher in virulent strains of *L. corymbifera* rather than in attenuated strains. The authors suggested that *L. corymbifera* uses the macrophages as vectors for dissemination in the blood and as protection from the immune system and antifungal agents [75]. Another study confirmed this finding and found that *Rhizopus conidia* stopped swelling inside the macrophages, so melanin is accumulated in the cell wall of the fungus. This melanin inhibits the phagosome maturation by continued induction of Akt/PI3K signaling [76].

The same effect of macrophages on germinating spores was observed in studying the effect of neutrophils, keeping in mind that neutrophils’ effect on pathogens is different from that of macrophages. In general, neutrophils produce catatonic peptides, induce oxidative burst and activate an inflammatory state in the body. In the case of *Rhizopus* infection, it was observed that its germinating spores (swollen) produce chemotactic factors to recruit the neutrophils via complement activation but the resting spores do not [77]. As a result, the neutrophils produce cationic peptides Np1 and Np2 to kill the swollen spores of *Rhizopus*, and no effect was observed on resting spores (same as macrophages effect) [77]. The neutrophils respond as well to *Rhizopus* hyphae by inducing proinflammatory gene expression, such as *TNF-a* and *IL-1b*, which leads to damage of the hyphae, but this damage is lower than that encountered with other fungi such as *A. fumigatus* [78]. Some studies pointed out that the neutrophil response toward *Rhizopus* hyphae is impaired in hyperglycemia, which further increases the risk of diabetic patients to mucormycosis [79].

The hallmark of mucormycosis is angioinvasion and local thrombosis at site of infection. Also, Chang *et al.* reported that thrombocytopenia is a major risk factor for developing mucormycosis in organ transplant patients [80]. All of these findings point out that platelets may have an effect in defending the body against invasive mucormycosis. Perkhofer *et al.* found that the platelets adhere to both the fungal hyphae and germinating spores and stop their elongation and germination, respectively [81]. NK cells induce cell cytotoxicity to lessen tissue damage upon receiving signals from macrophages, dendritic cells or T cells through secretion of IL-12 and type-1 interferon (IFN) [65]. In case of mucormycosis, the NK cells’ effect is highly pronounced toward *Rhizopus* hyphae not the conidia. The direct contact between the NK cells and the hyphae induce the production of perforin. However, it was found that the hyphae have immunosuppressive effects which decrease the production of IFN-γ and regulated on activation, normal T cell expressed and secreted (RANTES); CCL5 [82].

Although the role of adaptive immunity in fighting fungal infection is crucial, it plays a secondary role in case of Mucorales infection. Traditionally, T helper 1(T_H1_) produces IFN-γ to provide immunity against fungal infection but IL-4 produced by T_H2_ increases the susceptibility to infection. Moreover, IL-17 produced by subset of T_H1_, T_H17_, provides mucosal immunity against fungal infection. Furthermore, dendritic cells after recognizing β-glucan of the fungal cell wall produces IL-23, which allows the development of T_H17_ [83]. The activation of the pathway of IL-23/T_H17_ leads to induction of an inflammatory state in the body, which leads to neutrophils recruitment. In case of *Rhizopus* infection, its hyphae stimulates the production of IL-23 by dendritic cells [83].

## Risk factors

Patients at high risk for mucormycosis are those having preexisting comorbidities such as uncontrolled diabetes mellitus (DM), DKA, lung diseases, ventilator-associated pneumonia, neutropenia or high iron levels [84]. Patients on deferoxamine [85] or having hematological malignancies such as leukemia, lymphoma, multiple myeloma, myelodysplastic syndrome, aplastic anaemia and sideroblastic anaemia [86,87], undergoing stem cell transplants or organ transplant patients are also at risk [88]. Patients who use corticosteroids, tocilizumab and iatrogenic immunosuppression [89,90] may also develop mucormycosis. To a lesser extent, it was found that high doses of glucocorticoids can have a role in impairing the phagocytosis process and the intracellular killing of Mucorales spores [91].

There are other conditions that may increase the risk of developing mucormycosis, such as renal insufficiency, presenting with HIV or AIDS [92], the use of contaminated medical tools near or at open wounds, open skin trauma including burns or other injuries or cuts in skin [93]. Extreme malnutrition, the use of illegal drugs that involve needles, as well as hepatitis or cirrhosis have all been associated with mucormycosis [94].

Mucormycosis has even invaded the pediatric population [95]. Additionally, premature newborns can be at higher risk to develop mucormycosis infection as well [96].

## Clinical presentation

There are five major forms of infection that can be summarized as rhino-orbito-cerebral, pulmonary, gastrointestinal, cutaneous and disseminated infection, with the rhinocerebral presentation being the most common form [97]. The clinical manifestations of the rhino-orbital-cerebral form (which begins in the paranasal sinuses after the inhalation of Mucorales spores and expands to orbitals and brain tissues), include symptoms that are similar to complicated sinusitis, nasal blockage, headache, blackish or bloody nasal discharge, redness around the eyes and nose, facial pain with numbness, eye pain with blurry vision, ocular-motility changes, periorbital cellulitis, orbital inflammation and drooping eyelids [98–100]. Symptoms of intracranial invasion may present as neurological signs and altered mental status [101]. However, the patient may exhibit one or more of the listed signs and symptoms according to the severity of the disease [102].

The symptoms of the pulmonary type with the hyphal invasion of the pulmonary blood vessels include high-grade fever (>38°C), accompanied by nonproductive cough and airway obstruction. If the invasion has reached the hilar blood vessels, a massive hemoptysis may be present [103,104]. Meanwhile, the ingestion of the mucor spores may result in the gastrointestinal form of infection, which can show celiac, appendiceal, iliac or gastric perforation [105].

Surprisingly, the clinical manifestations of mucormycosis differs by epidemiology because different species inhabit different habitats, leading to different clinical profile. For instance, *Saksenaea* is predominant in North and South America and is known to cause cutaneous manfestations and, to a lesser extent, rhino-orbital symptoms. An interesting meta- analysis linking the epidemiology and clinical manifestation was studied recently [106]. The direct inoculation of the spores into the skin through trauma or burns in a susceptible host can show the clinical manifestations of the cutaneous form. This form of infection typically presents with black necrotic eschar and surrounding edema. The symptoms vary from localized disease to progressive fulminant disease. The latter may involve tissue gangrene and hematogenous dissemination [107,108].

Finally, patients with profound iron overload, immunosuppression, neutropenia and active acute leukemia are the classic group of patients at risk for the disseminated form [84,109–111]. The symptoms vary widely depending on the degree of dissemination and the vascular invasion [112].

## Diagnosis

The rapid dissemination of mucormycosis is believed to be an extraordinary phenomenon. A delay of only 12 h in the diagnosis can be lethal, and this is why 50% of the mucormycosis cases are diagnosed only in *postmortem* autopsy [113]. Chamilos *et al.* have shown that the delayed initiation of the treatment has resulted in twofold increase of mortality rate in the 12-week post-diagnosis assessment, compared with early treatment initiation (82.9% vs 48.6%) [114]. Indeed, a high index of suspicion is needed to make the appropriate diagnosis and initiate prompt treatment. The diagnosis can be categorized into probable infection, possible infection and proven infection.

Probable infection of invasive mucormycosis includes the involvement of a host factor (presence of neutropenia, more than 3 weeks of corticosteroids, use of immunosuppressors, presence of hematological malignancies etc.), presence of a clinical criterion (imaging reveals lower respiratory tract infection, signs for sinusitis or sinonasal infection, bronchitis etc.), a mycological criterion by direct or indirect techniques (cytology, microscopy, culture, immune-detection of antigen or cell wall components).

Possible infection of invasive mucormycosis includes cases that comply with both the criteria for a host factor and a clinical criterion [115]. Meanwhile, the diagnosis of a proven mucormycosis can be done through histopathologic, cytopathologic or direct microscopic investigation, illustrating the fungal hyphae (nonseptate or pauci-septate, of width of 6–16 μm) in the biopsy specimen, along with accompanied tissue damage, mycotic infiltration of blood vessels, or positive culture results [116]. Both focal bony erosions with the extrasinus spread are strongly indicative for the diagnosis of mucormycosis [56].

PCR could be the diagnostic modality for molecular identification of the organism [117]. The conventional radiological techniques are not specific for the diagnosis of mucormycosis. In contrast, the diagnosis of mucormycosis includes computed tomography (CT) of the chest; identifying the infiltrates that are not documented by the standard or regular radiograph [118]. The use of noncontrast CT of the paranasal sinuses is often the investigation of choice. MRI of orbit, brain and paranasal sinuses can assess the extent and the severity of disease [102]. In cases of intracranial or intraorbital spread, gadolinium-enhanced MRI is used [56].

To confirm the diagnosis of mucormycosis, laboratory methods for identification of the organisms and their culture are carried out [119]. Direct visualization of the organisms can be done by wet mount of KOH using bright microscope or fluorescent microscope after staining them with Blankophor and Calcofluor White fluorescent stains [120]. Morphologically, the hyphae of Mucorales are nonseptate rather than the septate hyphae of *Aspergillum* [119]. They have a characteristic ribbon-like structure with a pattern of branching from 45° to 90°. Mucorales hyphae are weakly stained with Gomori methenamine silver and periodic acid–Schiff techniques due to its thin wall [121]. These fungi grow normally on Sabouraud agar for 3–5 days at 25–30°C. Microaerophilic conditions similar to infracted tissues are required when culturing *Cunnighamella* and *Rhizopus* [120]. There are many negative culture results despite positive microscopic identification due to possible mechanical damage of hyphae during preparation of the sample. However, better culture results were obtained at 37°C [122]. Although Mucorales cause angioinvasion, the blood cultures are negative [123]. In case of neutropenic or immunocompromised hosts, the positive finding from the bronchoalveolar would suggest the presence of the infection and should mandate initiation of treatment [124,125]. However, the histological examination of tissues from biopsies is the method of choice for the diagnosis. The invasion been seen on the histopathology is necessary to confirm the diagnosis with mucormycosis [126]. However, current diagnostic tools may fail to provide rapid results. Recently, there has been a trend for the use of rapid micro-culture assay strategies for early diagnosis of Mucorales infections caused by *R. arrhizus* directly in blood and tissue samples [127].

## Treatment

Early diagnosis and treatment is crucial because rapid progression of disease and higher mortality rate from intraorbital and intracranial complications can reach (50–80%) [128]. However, even with the prompt diagnosis and immediate treatment of the underlying diseases, along with the aggressive medical/surgical interventions, the management is still not fully effective. This eventually may lead to the spread of the infection and ultimately higher mortality rates [56].

Although the uncontrolled DM is a major risk factor for mucormycosis, diabetic patients may have a better outcome when it comes to rhino-orbito-cerebral mucormycosis than nondiabetic patients, as reported by Yohai *et al.* and Biltzer *et al.* [129,130]; those with leukemia and lymphoma, however, are believed to have poorer outcomes [94].

Starting with treating the patient’s underlying medical condition and tapering the immunosuppressive agents are the first steps toward treatment. The mainstay of treatment is surgical removal of the infected parts along with the use of systemic antifungal agents. However, the choice of the proper antifungal agent seems to be limited because the Mucorales are inherently resistant to most popular and widely used antifungals [131,132].

The treatment recommendations provided in this review are supported by the 2019 global guidelines for the diagnosis and treatment of mucormycosis, by the ECMM and the Consortium for Mycosis Education and Research [133], providing more detailed guidance on management and alternative therapeutic options for mucormycosis [119].

The guidelines generally support prompt, early and complete surgical debridement of the infected area whenever possible. The start of a systemic antifungal treatment is crucial as well. There's a 1.5-fold increase in the survival rates when combining surgical interventions with early, high doses of systemic antifungal agents [114,134]. Surgical approach alone was reported to be not curative; however, aggressive surgical interventions were shown to have better survival rate [57,135].

According to the global guideline for the diagnosis and management of mucormycosis in 2019 [119], the first-line antifungal monotherapy agent is liposomal amphotericin B, with a dose of 5–10 mg/kg/d. However, when substantial renal toxicity develops, a reduction in the dose can be done as necessary. When there is brain involvement or solid organ transplant, the dose should be 10 mg/kg/d, initiated from day 1. Doses below 5 mg/kg/d are marginally recommended [118,136]. Doses up to 15 mg/kg/d were proven by Walsh *et al.*, to be well tolerated [137]. Amphotericin B lipid complex with dose of 5 mg/kg/d is recommended in patients presented with CNS involvement [119]. Amphotericin B is a polyene that exerts its antifungal effect via binding to ergosterol, providing the structure and rigidity of the fungal cells. Amphotericin B forces the cell membrane to leak and eventually leads to cell death [138].

For decades, amphotericin B deoxycholate has been the drug of choice [4,134]. Despite its effectiveness, its use was limited due to its substantial toxicity [139,140]. Current guidelines recommend against its use with the exception of settings where there is no other available antifungal therapy.

Triazoles, the largest class of antifungal agents in the clinical practice, can be added as well. They act on inhibiting the 14-α-demethylation, which in turn can lead to an increase in the toxic 14-α-methylsterols, the one that alters the permeability of the fungal membrane [141]. Isavuconazole, a broad-spectrum antifungal, with less hepatotoxcity than other mold-active azoles, has been licensed by USA as the first-line treatment of mucormycosis [142].

The approved dose for isauvoconazole as a treatment for mucormycosis is 3 × 200 mg to be given for day 1–2, and 1 × 200 mg to be given from day 3. A dose of 372 mg of isavuconazonium sulfate (the currently available prodrug form of isauvoconazole) is equivalent to 200 mg isavoconazole [143]. Isavoconazole is also strongly recommended as salvage treatment with proven activity in clinical scenarios, refractory disease, intolerance or toxicity [143,144].

Posaconazole, a second-generation triazole, is advised to be used prophylactically in high-risk patients such as neutropenic patients or those with grafts. Posaconazole is considered as a salvage therapy for patients who cannot withstand or intolerant to the amphotericin B [145]. Until recently, posaconazole was only provided in suspension form for the management of invasive fungal infections with respect to the long-term use. Unfortunately, the suspension form has displayed variable pharmacokinetics. Drug concentration was reported to be less than predicted at the target site [141].

However, the introduction of delayed-release tablet form of posaconazole has succeeded to address most of the absorption concerns The dose of posaconazole’s tablet for the treatment of fungal infections is 300 mg orally twice on the first day, followed by 300 mg orally once daily, regardless of food timing [60]. The intravenous formulation can be provided for oral intolerant patients; the dosing regimen is the same as the delayed-release formulation.

The use of combination therapy can be under the umbrella of lack of enhanced toxicity with possible but unproven added benefit. There is no definitive data to guide the use of antifungal combination therapies, and they are kept as marginal recommendation. However, the use of combination antifungal agents was addressed in large number of studies. Although the echinocandins have no inherent activity against mucormycosis, some evidence suggests that echinocandins may augment the polyene therapy. The benefit of adding the echinocandins is to provide a polyene backbone that enhances and augments therapy. They are believed to inhibit the β-1, 3-glucan, a cell-wall component [146]. In a retrospective review of two institutions, enhanced outcomes were shown by the use of a combination of polyene–caspofungin therapy in rhino-orbital and rhino-orbital-cerebral patients, compared with polyene-only therapy [147]. On the other hand, the use of deferosirox (iron chellator) with liposomal amphotericin B revealed a higher mortality rate of patients at day 90, hence, it is not recommend to use adjunctive deferasirox as a part of initial combination regimen [147].

Hyperbaric oxygen has been proposed as a beneficial adjunctive treatment for mucormycosis, especially in patients with diabetes [148]. High concentration of oxygen has a fungicidal activity and can inhibit the growth of Mucorales *in vitro* [149]. It can also improve the neutrophil activity, supply better flow of oxygen to ischemic tissues and improve wound healing [148,150,151]. In a previous study, the use of standard therapy of mucormycosis had a 22% survival rate compared with 83% survival rate in patients who have received standard therapy with hyperbaric oxygen [150]. However, the use of hyperbaric oxygen is limited due to its only experimental and limited clinical data.

Both interferon and granulocyte-macrophage colony-stimulating factor act as enhancers to the granulocytes’ ability to damage the Mucorales [152]. The treatment with recombinant granulocyte colony-stimulating factor (G-CSF) has been used in combination with lipid amphotericin B, showing promising outcomes [153–155]. The use of G-CSF-mobilized granulocyte transfusions has been used in refractory mycoses, including mucormycosis [156]. However, data regarding their use is limited.

Unfortunately, despite aggressive surgical intervention and the use of systemic antifungals, poor prognosis and high mortality rates (33.8–80%) has been reported in disseminated infections [157,158]. Unfortunately, mucormycosis can lead to eyes and upper jaw loss. Patients should be psychologically prepared to face and accept the loss of function that comes, for example, with a missing jaw, as chewing problems, swallowing difficulties and facial aesthetics. In patients who lose their eyes or upper lip, mechanical substitution or prosthetic constructions are considered to be options.

## Mucormycosis & COVID-19

COVID-19 continues to cause significant fatalities worldwide. Until the emergence of an effective antiviral therapy, glucocorticoids have proved to play essential roles in COVID-19 management – namely, reducing mortality in hypoxemic COVID-19 patients [159]. Yet the extensive use of steroids/monoclonal antibodies such as tocilizumab/broad-spectrum antibiotics together with the virus immune dysregulation may all together exacerbate previous fungal diseases and can upsurge the danger of secondary infections in COVID-19 patients [160–163].

Although previously of low incidence rate, numerous mucormycosis cases have been reported recently as a consequence of the COVID pandemic, leading to a substantial rise in its incidence [164]. Unfortunately, a definite correlation between COVID-19 and elevated mucormycosis infections is obvious. For example, a teaching hospital in India has reported 23 cases of sinus mucormycosis in only 4 months, with all patients being COVID-19 positive [165]. Mehta and Pandey reported a case of COVID-19 associated rhino-orbital mucormycosis in September 2020 [160]. Another report was issued by Werthman-Ehrenreich that month [164]. In another study, Garg *et al.* reported three subjects with COVID-19-associated mucormycosis lacking the traditional risk factors, such as DM, transplantation or hematological malignancies, which was one worrying finding. The development of mucormycosis was probably due to the use of glucocorticoids and hence, the utilization of higher doses of glucocorticoids should be prevented [166]. Steroid therapy indeed appears to be a double-edged sword, predisposing patients to secondary bacterial and invasive fungal infections, thus impacting morbidity and mortality [167]. Pandiar *et al.* recently hypothesized that the COVID-19 generates an environment for proliferation of Mucorales and consequent mucormycosis. In their study, they proposed a new hypothesis for the incidence of mucormycosis with scientific proof that explains dysregulation of ACE-2 expression in lungs and several tissues and how this results in a cascade of pathways that creates a suitable milieu for mucormycosis. Hence, COVID-19 infected patients are at a higher risk of contracting mucormycosis [168].

In a new systematic review carried out through 13 May 2021, 101 cases of COVID-19-associated mucormycosis have been reported, including 82 cases from India and 19 from elsewhere. Mucormycosis was principally perceived in males (78.9%). Hyperglycemia at presentation was the central predisposing factor detected in most cases (83.3%), followed by cancer (3.0%). Preexisting DM accounted for 80% of cases, while associated DKA was present in ~15% of cases. Steroid intake was reported in 76.3% of cases, followed by remdesivir (20.6%). The most common organ involved with mucormycosis was nose and sinus (88.9%), followed by rhino-orbital (56.7%) and ROCM type (22.2%). Total mortality was recorded as 30.7% of the cases. Jointly, these observations imply that COVID-19, diabetes and steroids is a dreadful triad in mucormycosis patients [169]. In addition, this study revealed the serious problem facing India. Rising cases of COVID-19 associated mucormycosis especially in India may be due to the very high incidence rate of type 2 diabetes and the large proportion of people who do not receive health care or do not undergo diagnostics. Moreover, their overloaded hospitals further enhance the spread of mycoses.

In this review, we have reported the appearance of additional COVID-19 associated mucormycosis cases from India. A few days ago, Krishna *et al.* reported a post-COVID-19 patient in India, who was an uncontrolled type 2 diabetic male and was diagnosed with mucormycosis of the right maxilla [39]. Nehara *et al.* reported the first case series of COVID-19 associated mucormycosis patients from India consisting of five cases of COVID-19 infection, who developed rhino-orbital mucormycosis during their treatment [170]. Numerous other cases are being described in electronic media in different countries, however, not officially reported.

Indeed, the situation in India is critical, where both COVID-19 and mucormycosis are considered a double health threat to their healthcare system. The use of corticosteroids in COVID-19 patients with diabetes mellitus is lifesaving [171]. On the other hand, it makes the room for worsening the diabetic control, creating the ultimate environment for opportunistic infections such as mucormycosis. Adding to the immunosuppressant nature of glucocorticoids they may also provide another foothold for invasion [172]. Proper glycemic control, wise use of glucocorticoids, prompt antifungal treatment with proper surgical debridement are the recommended patterns for management [7].

A sum of factors may trigger mucormycosis in COVID-19 patients. The principal reason enabling Mucorales spores to propagate in COVID-19 individuals is a perfect setting of hypoxia. COVID-19 also leads to endothelialitis, endothelial damage, thrombosis, lymphopenia and reduction in Mucorales-specific T cells (CD4+ and CD8+). These T cells produce cytokines including IL-4, IL-10 IL-17 and IFN-γ, which impair the fungal hyphae thus their reduction predisposes to secondary fungal infection [173]. In addition, the existence of DM or DKA intensifies the danger of developing mucormycosis due to hyperglycemia and because it aggravates COVID-19 severity. Steroid therapy to treat COVID-19 can also cause hyperglycemia, even in healthy individuals, and cause steroid induced diabetes. In addition, steroid therapy together with DM can enhance immunosuppression and hyperglycemia, elevating the infection risk [174]. DKA is also often detected due to steroid consumption. Decreased pH caused by acidosis creates a suitable media for mucor spores germination. Further, steroids decrease the phagocytic activity of white blood cells and impair bronchoalveolar macrophages migration and ingestion, making the diabetic patient extremely susceptible to mucormycosis [169]. Furthermore, hyperglycemia results in glycosylation of transferrin and ferritin, which decreases iron binding, providing free available iron as an ideal resource for mucormycosis. Increased cytokines such as IL-6 also increases free iron by increasing ferritin levels due to increased synthesis and decreased iron transport. Acidosis further increases free iron by the same mechanism and also decreases the transferrin ability to chelate iron [175]. Finally, lengthy hospital stays with or without mechanical ventilators may also predispose to the development of mucormycosis.

## Recommendations

It is recommended to avoid the extensive overzealous use of steroids and wide-spectrum antibiotics in the treatment course of COVID-19. SARS-CoV-2 spikes glycoproteins were recently investigated for designing potential antiviral targets [175]; however, drugs targeting immune pathways (e.g., tocilizumab) should be prevented if not clearly beneficial. These agents should be monitored to achieve their therapeutic response at the lowest dose, and over the shortest duration possible. As there is a growing evidence about confirmed new cases of mucormycosis that has been linked to COVID-19, there is a need for better awareness regarding the importance of early identification, fast diagnosis and prompt treatment initiation, which may significantly reduce the morbidity and mortality rates. However, a major obstacle faced by clinicians is how to choose among the current available antifungal agents in the treatment of COVID-19-associated mucormycosis given the lack of active clinical trials.

For efficacious management of mucormycosis, synchronized tasks from an integrative group including infectious diseases, ophthalmology, neurosurgery, otorhinolaryngology, pathology and microbiology sections are vital. The Indian Council of Medical Research has suggested that doctors should carry out basic tests such as sinus tenderness, vision and ocular motility in the physical assessment of severely ill or diabetic COVID-19 patients or those using steroids. Particular care should be given to patients showing mucormycosis signs including one-sided nasal obstruction or headache, sinus pain, swelling, numbness or toothache [176].

It is important to spread awareness among both the healthcare system and the general public regarding how serious and fatal the mucormycosis infection is. It is crucial to educate the patients to report their symptoms as early as possible so they can help the healthcare team to have prompt onset of diagnosis of the infection. COVID-19 patients or those who have just recovered from COVID-19 infection should try not expose themselves to any of the natural habitats of the fungus. Mass vaccination against COVID-19 will also help to reduce the probability of severe or secondary complications. Hospitals have to ramp up their hygiene level, preventing the onset of damp areas that might be a breeding ground for mucormycosis. Caution should be taken particularly when dealing with oxygen cylinders, ventilators, and breathing pumps.

## Conclusion

Mucormycosis seems to be associated with the COVID-19 pandemic and role of the immune response in disease progression. The extensive use of steroids and wide spectrum antibiotics in the treatment course of COVID-19 should be avoided or at least closely monitored. Drugs targeting immune pathways should be prevented if not clearly beneficial and they should be monitored to achieve their therapeutic response at the lowest dose. There is a growing evidence about confirmed new cases of mucormycosis that has been associated with COVID-19. There is a need for better awareness regarding the importance of early identification, fast diagnosis and prompt treatment initiation of mucormycosis particularly, under the lack of active clinical trials.

## Future perspective

The collection of epidemiological data is important for taking appropriate and affordable measures against mucormycosis infection. Guidelines should be developed and implemented in all healthcare facilities to enable epidemiological data collection and rapid reporting of any outbreaks. Antifungal agents stewardship programs should be implemented for antifungal prescription and use as well as for the control and monitoring of infections caused by the clinically relevant pathogens in healthcare facilities. Rational use of corticosteroids as well as immunosuppressive agents should be undertaken and monitored. Alternative approaches for treating such infections should be considered, such as phage therapy or promising combination of antifungal agents. Finally, strict and protective measures must be taken to prevent dessimanation of this life-threatenging fungal pathogen.

**Executive summary**

- Mucormycosis seems to be associated with the COVID-19 pandemic.
- Historical records, taxonomy and epidemiology of mucormycosis are illustrated here.
- There are multiple risk factors of mucormycosis; patients with comorbidities or having hematological malignancies are on the top of the list.
- There are five major forms of infection that can be summarized as rhino-orbito-cerebral, pulmonary, gastrointestinal, cutaneous and disseminated infection, with the rhinocerebral presentation being the most common form.
- Early diagnosis and treatment is crucial.
- Steroid therapy appears to be a double-edged sword in the era of COVID-19.
- COVID-19 associated mucormycosis in now creating an epidemic in a global pandemic, particularly in the low- to middle-income countries.
- It is important to spread the awareness among both healthcare system and general public regarding how serious and fatal the mucormycosis infection is.
